# Monitoring integrity and localization of modified single-stranded RNA oligonucleotides using ultrasensitive fluorescence methods

**DOI:** 10.1371/journal.pone.0173401

**Published:** 2017-03-09

**Authors:** Philipp Heissig, Waldemar Schrimpf, Philipp Hadwiger, Ernst Wagner, Don C. Lamb

**Affiliations:** 1 Pharmaceutical Biotechnology, Department of Pharmacy and Center for NanoScience (CeNS), University of Munich (LMU), München, Germany; 2 Physical Chemistry, Department of Chemistry and Center for NanoScience (CeNS), University of Munich (LMU), München, Germany; 3 Axolabs GmbH, Kulmbach, Germany; Consiglio Nazionale delle Ricerche, ITALY

## Abstract

Short single-stranded oligonucleotides represent a class of promising therapeutics with diverse application areas. Antisense oligonucleotides, for example, can interfere with various processes involved in mRNA processing through complementary base pairing. Also RNA interference can be regulated by antagomirs, single-stranded siRNA and single-stranded microRNA mimics. The increased susceptibility to nucleolytic degradation of unpaired RNAs can be counteracted by chemical modification of the sugar phosphate backbone. In order to understand the dynamics of such single-stranded RNAs, we investigated their fate after exposure to cellular environment by several fluorescence spectroscopy techniques. First, we elucidated the degradation of four differently modified, dual-dye labeled short RNA oligonucleotides in HeLa cell extracts by fluorescence correlation spectroscopy, fluorescence cross-correlation spectroscopy and Förster resonance energy transfer. We observed that the integrity of the oligonucleotide sequence correlates with the extent of chemical modifications. Furthermore, the data showed that nucleolytic degradation can only be distinguished from unspecific effects like aggregation, association with cellular proteins, or intramolecular dynamics when considering multiple measurement and analysis approaches. We also investigated the localization and integrity of the four modified oligonucleotides in cultured HeLa cells using fluorescence lifetime imaging microscopy. No intracellular accumulation could be observed for unmodified oligonucleotides, while completely stabilized oligonucleotides showed strong accumulation within HeLa cells with no changes in fluorescence lifetime over 24 h. The integrity and accumulation of partly modified oligonucleotides was in accordance with their extent of modification. In highly fluorescent cells, the oligonucleotides were transported to the nucleus. The lifetime of the RNA in the cells could be explained by a balance between release of the oligonucleotides from endosomes, degradation by RNases and subsequent depletion from the cells.

## Introduction

Oligonucleotide therapeutics have gained in importance over the last decades as they can be utilized to interfere with almost every cellular process by simply selecting the appropriate sequence and format[1]. Combined with the progress that has been made in the delivery of oligonucleotides, some of them are already approved for market access and numerous candidates are currently under investigation in clinical trials for treatment of a variety of different diseases[2, 3]. Delivery can be accomplished in both the double or single-stranded configuration[4]. While the double-stranded representatives are mostly limited to RNA interference[5, 6], single-stranded oligonucleotides have a broader spectrum of applications. Single-stranded microRNA[7, 8] or siRNA guide strands[9–11] have been shown to successfully mediate RNA interference and antagomirs have been used to effectively down regulate endogenous microRNAs[12, 13]. CpG oligodeoxynucleotides are single-stranded DNA oligonucleotides containing an unmethylated cytosine/guanine motif, which acts as an immunostimulant through the Toll-like receptor 9[14, 15]. Furthermore, antisense oligonucleotides represent a class of complementary sequences that can interfere with mRNA at various processing stages including splicing, translation or polyadenylation[16, 17]. Compared to double-stranded RNAs, the single-stranded formats are more prone to nucleolytic degradation upon exposure to the cellular environment. Chemical modification of the RNA backbone has proven to be an attractive solution to slow down or even suppress nucleolytic degradation. Examples include ribose modifications in the 2’ position such as 2’-O-Methyl, 2’-Fluoro or locked nucleic acids (LNA) [18, 19]. Another highly nuclease protective intervention, especially in combination with the 2’-modifications mentioned above, is the replacement of the natural phosphodiester linkages by phosphorothioates where one of the non-bridging oxygens is replaced by sulfur[20]. Most studies on the impact of chemical modifications on bioactivity rely on quantitative read-out systems like reporter gene knockdown efficiency[21, 22] or, in the case of antagomirs, on microRNA target up regulation[23, 24]. Less focus has been placed on the intracellular fate of such modified RNAs. Examples include a study indicating the formation of nuclear bodies after transfection of phosphorothioate oligonucleotides[25] and a work on subcellular trafficking of modified molecular beacons by fluorescence microscopy[26]. Furthermore, the behavior of differently modified antagomirs in mice was investigated by Stoffel and coworkers[27] and an interesting study by Hirsch *et al*. examined the duplex stability and localization of siRNA by intensity based FRET[28].

In this work, we measured the stability of different chemically modified, short, fluorescently-labeled RNA oligonucleotides using fluorescence spectroscopy and microscopy methods. In cellular extract, we measured the stability of the various constructs using fluorescence correlation spectroscopy (FCS), fluorescence cross-correlation spectroscopy (FCCS) and Förster resonance energy transfer (FRET). The RNAs consisted of different chemically stabilizing modification patterns and were labeled with a FRET pair (Atto488 and tetramethylrhodamine (TMR)). Their stability correlated with the extent of chemical modification. Utilizing the different read-outs of each technique made it possible to distinguish degradation from unspecific effects. For measurements in cultured cells, we present an approach to monitor the localization and integrity of such small RNAs using fluorescence lifetime imaging microscopy (FLIM). Transfection was accomplished with a sequence defined cationic transfection agent recently developed in our lab[29, 30]. As the lifetime of the donor dye increases with decreasing FRET, each spot in the cell can be assigned a lifetime value representing the integrity of the RNA at this specific location. The extent of chemical modification correlated with RNA integrity and with an increasing intracellular accumulation over time. For highly transfected cells, we observed a significant nuclear translocation of the RNAs.

## Methods

### Coupling of the RNA oligonucleotides to Atto488 and tetramethylrhodamine

The 3’-amino and 5’- C6SSC6 disulfide modified oligonucleotide (Axolabs, Kulmbach, Germany) was dissolved in 100 mM sodium borate buffer containing 20% acetonitrile (pH 8.5) to a final concentration of 800 μM. Atto488-NHS ester (Atto-Tec, Siegen, Germany) was dissolved in anhydrous DMSO to a working concentration of 1 mM. Three molar equivalents of Atto488-NHS ester solution were added over 2 h every 15 min, following 3 h incubation at 25°C. The resulting construct was purified by EtOH precipitation and redissolved in water to a concentration of 1 mM. The C6SSC6 disulfide modified end was reduced with buffered tris(2-carboxyethyl)phosphine (TCEP, 700 times molar excess, Sigma Aldrich, Steinheim, Germany) for 2.5 h at RT. TCEP was removed by EtOH precipitation. The remaining pellet was redissolved in 50 mM sodium phosphate buffer 20% acetonitrile (pH 7) to a concentration of 800 μM. Tetramethylrhodamine-6-maleimide (Life Technologies, Darmstadt, Germany) was dissolved in anhydrous DMSO to a working concentration of 1 mM. The tetramethylrhodamine-6-maleimide solution (1.3 equivalents) was added immediately to the oligonucleotide solution, following incubation of 2 h at 25°C. The product was purified by EtOH precipitation and high-performance liquid chromatography.

## Purification with high-performance liquid chromatography

Purification of the dual-labeled RNA oligonucleotide was performed using high-performance liquid chromatography (VWR Hitachi Chromaster consisting of 5430 Diode array detector and 5160 gradient pump, Darmstadt, Deutschland). The products were separated with a XTerra C8 column (5 μm, 4.6 x 150 mm, Waters, Eschborn, Germany) and eluted with an ACN /0.1 M triethylammonium acetate gradient (5:95 to 65:35 in 30 min). Product containing fractions were lyophilized and stored at -20°C.

### Polyplex formation

The sequence-defined lipo-oligomer ***278*** was synthesized by solid-phase assisted synthesis as described in our previous publications [11, 29, 31]. It is a U-shaped lipo-oligocation consisting of a protonable backbone of three *succinoyl*-tetraethylene pentamine (Stp) units for complexation of the nucleic acids. Terminal cysteines introduced for polyplex stabilization by disulfide formation are separated from the Stp units by branching lysines, which are connected to four linoleic acids, which aid particle stabilization through hydrophobic interactions. The oligonucleotide and the required amount of oligomer ***278*** were separately diluted in 20 mM HEPES-buffered 5% glucose pH 7.4 in a final volume of 25 μL. Both solutions were pooled resulting in an amine/phosphate ratio of 20 (final concentration oligonucleotide: 1 μM, final concentration oligomer ***278***: 46 μM) and incubated for 45 min at RT.

### Cell culture

HeLa wild-type cells (ATCC, CCL-2) were cultured at 37°C in folate free RPMI 1640 medium, supplemented with 10% fetal calf serum, 4 mM glutamine, 100 U/mL penicillin and 100 μg/mL streptomycin (Life technologies, Darmstadt, Germany). For maintenance, the cells were detached with a trypsin-EDTA solution (0.25%) every two days and seeded at a dilution of 1/10.

### Transfection

HeLa wild-type cells were seeded in 8 well Nunc Lab-Tek chamber slides (Thermo Scientific, Germany) in 300 μL medium (25,000 cells per well). After 24 h, the medium was exchanged with 250 μL fresh medium. The formed polyplexes containing the oligonucleotide were added in a volume of 50 μL to each well resulting in an oligonucleotide concentration of 167 nM. After 15 min incubation time at 37°C, the medium was exchanged with 300 μL fresh medium. Fixation was accomplished by washing the wells twice with PBS (resuspending) at the desired time point (15 min, 1 h, 6 h or 24 h after transfection), followed by 10 min incubation with 4% paraformaldehyde/PBS at RT. The wells were washed three times with PBS and stored at 4°C up to 3 days.

### HeLa whole cell extracts

Cells were seeded in 150 cm^2^ plates. After 48 hours, the cells were detached with trypsin-EDTA solution (0.25%) and washed three times with PBS. The resulting pellet was resuspended in 4 packed cell volumes of buffer A (10 mM HEPES, 10 mM KCl, 1.5 mM MgCl_2_, 0.2 mM phenylmethylsulfonyl fluoride (PMSF), 0.5 mM dithiothreitol (DTT), pH 7.9) and sonicated three times for 5 s at 30% amplitude with 30 s incubation on ice in between. PMSF and DTT were added to the buffer immediately before use. The cells were centrifuged for 20 min at 14000 × g and the supernatant was collected. After aliquotation, the extract was frozen in liquid nitrogen and stored at -80°C.

### Cell extract measurements

HeLa whole cell extracts were incubated with 100 nM of the oligonucleotide at 37°C. The concentration of cell extract was optimized to ensure an appropriate degradation rate for a measurement duration of 3 h (1/10 dilution). Correlation and FRET measurements in cell extract were performed on a home-built pulsed interleaved excitation laser scanning confocal microscope described previously[32, 33] in TM buffer (10 mM Tris-Cl; 5 mM MgCl_2_; pH 7.5). For focusing the excitation light and collecting the fluorescence, a 60x water immersion objective with a numerical aperture (NA) of 1.27 was used (Plan Apo IR 60x WI, Nikon). This resulted in a diffraction limited lateral focus size *ω*_*r*_ of 210 nm for the green and 260 nm for the red channel, respectively. The laser power measured directly before the objective was set to 10 μW for the blue 475 nm laser and 3 μW for the yellow 565 nm laser. To prevent evaporation of the immersion liquid, an immersion oil with a refractive index of 1.33 was used.

During the measurements, the fluorescence intensity of the two channels was recorded at a single point in the solution. The experiments were performed at 37°C for 3 h each, divided into individual measurements of 1 min. A home written software package, PAM, was then used for FCS, FCCS and FRET analysis. The analysis methods are described in greater detail in the supporting information.

### FLIM measurement on fixed cells

FLIM measurements on fixed cells were performed on the same microscope as the cell extract measurements. For single cell images, a 1.27 NA 60x water immersion objective (Plan Apo IR 60x WI, Nikon) was used. Areas of 100 μm by 100 μm were recorded as 300x300 images, resulting in a pixel size of 333 nm. In order to image larger areas, a 0.45 NA 10x air objective was used (CFI Plan APO 10x 0.45 NA, Nikon). This resulted in 600 μm by 600 μm sized images with a pixel size of 1.17 μm for a resolution of 512x512 pixels.

For each region, 50–100 frames were recorded at a frame time of 5 s. The laser power of the 475 nm laser was set to 2–10 μW for the 60x objective and 10–90 μW for the 10x objectives to achieve a count rate between 50 kHz and 1 MHz. This guaranteed a high enough signal for the FLIM analysis while preventing artifacts from detector dead-time and photon pileup. The home written software package PAM was used for the phasor analysis of the FLIM data. A detailed description of the analysis method is given in the supporting information.

## Results & discussion

### Oligonucleotide design

**Fig 1:**
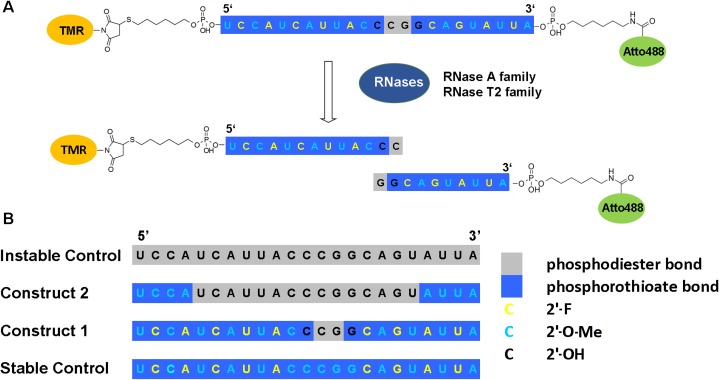
Design of the dual-labeled RNA oligonucleotide. (A) 23 nucleotide RNA oligonucleotide conjugated to tetramethylrhodamine (TMR) at its 5’ end *via* a thioether bond and to Atto488 at its 3’ end *via* an amide bond. Upon exposure to the cellular environment, the oligonucleotide can be degraded by various RNases. (B) Modification patterns selected to monitor intracellular localization and integrity of the oligonucleotide. RNA backbone modifications to modulate stability towards nucleolytic degradation: 2’-F, 2’-O-Me and phosphorothioate.

Our model sequence is 23 nucleotides in length, with a thiol modification at its 5’ and an amine modification at its 3’ end. The sequence was originally selected as a putative antagomir against microRNA200c, which has been the subject of other research in our lab [23, 34–35]. Loss of microRNA200c correlates with carcinoma progression to a more aggressive and chemoresistent mesenchymal state [23, 35–36]. The current study focuses on the stability and localization of the RNAs irrespective of their effector function and builds that basis for future sequence-specific degradation studies. Thus, to avoid any sequence-specific interactions, we have selected HeLa cells where microRNA200c is not present in significant quantities[37]. Atto488 (excitation maximum: 501 nm; emission maximum: 523 nm, NHS-ester modified) and TMR (excitation maximum: 557 nm; emission maximum: 576 nm, maleimide modified) were selected as a FRET pair for dual labeling of the RNA constructs at the ends and attached to the 3’ end *via* an amide bond and to the 5’ end *via* formation of a thioether. This approach provided us with a very sensitive read-out as cleavage of a single nucleotide already leads to separation of the two dyes (Fig 1A). Cleavage can be detected using FCS (through a reduction in the diffusion time), FCCS (*via* a loss in cross-correlation signal) and FRET (*via* a loss in FRET signal, which can be detected using either fluorescence intensity or fluorescence lifetime measurements). After the sequence is exposed to the cellular environment, cleavage is mediated by nucleases. RNase A and RNase T2 family members cleave single-stranded RNA with high specificity and affinity[38, 39].

Three different chemical backbone modifications were selected for our approach: natural phosphodiester bonds were replaced with phosphorothioate bonds (PS). RNA residues were replaced by 2’-modified analogues. We selected 2’-F and 2’-O-Methyl modifications as these are widely used in RNAi and antisense applications. As a stable control, a sequence consisting of a completely PS-modified backbone and alternating 2’-O-Me and 2’-F modifications was selected, since it was shown that this sequence provides adequate nuclease resistance[9]. Construct 1 is almost identical to the stable control with the exception of a single phosphodiester bond between nucleotide 10 and 11 counted from the 3’ end surrounded by two non-modified nucleotides on each side (Fig 1B). This provides a relatively stable sequence, which might still be susceptible to nucleolytic cleavage and can be used to investigate the intracellular location-specific RNase activity. Construct 2 has four PS and four 2’-O-Me modifications at the 5’ and the 3’ ends, giving a stretch of 15 unmodified nucleotides in the middle. This should result in faster nucleolytic degradation, however, leaving the dyes attached to a four-nucleotide RNA strand. Hence, the cell treats the residual, labeled construct similar to unlabeled RNA even after degradation of the unmodified domain. As a second control, a completely unmodified RNA strand is included to confirm the effect of the modifications (Fig 1B).

### Stability evaluation in cell extracts

Initial experiments were conducted in cell extracts to get a first hint of the stabilizing effect of the modifications. The time course of degradation was observed for 3 h every 5 min with a confocal microscope. Evaluation was accomplished simultaneously using FCS, FCCS and FRET, providing us with distinct information about the behavior of the differently modified oligonucleotides.

#### Fluorescence correlation spectroscopy

**Fig 2:**
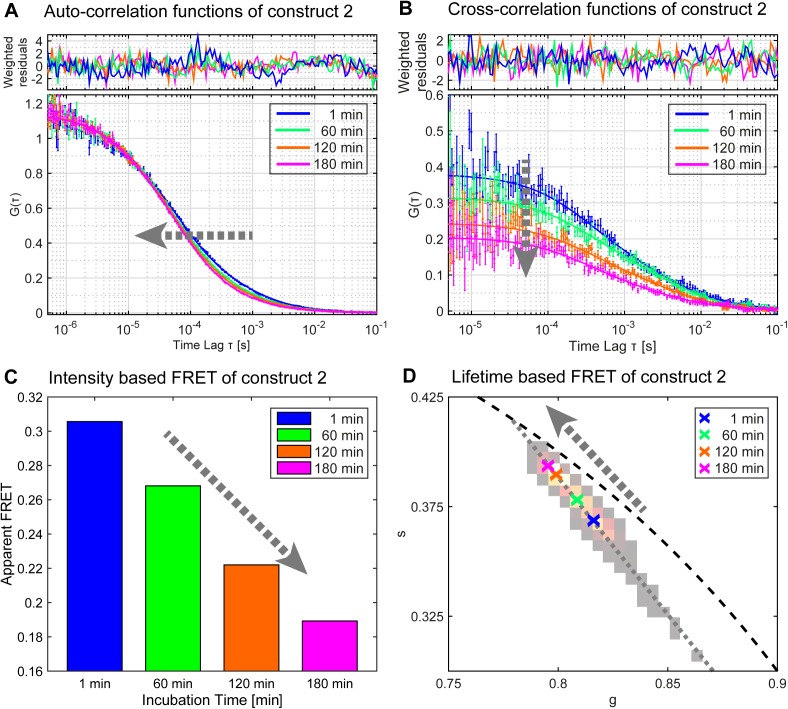
Monitoring oligonucleotide degradation using FCS, FCCS and FRET. The stability of various RNAs was measured as a function of incubation time in cell extracts. The main changes and parameters corresponding to RNA degradation are shown exemplary for construct 2, representing: (A) the diffusion time from the autocorrelation function (FCS), (B) the amplitude of the cross-correlation function (FCCS), (C) an apparent FRET efficiency determined from the fluorescence intensity and (D) the donor fluorescence lifetime based FRET using a phasor analysis. The colored crosses represent the center of mass in the phasor plot of measurements after 1 min (blue), 60 min (green), 120 min (orange) and 180 min (magenta). The grey arrows indicate the direction of the main changes.

The diffusion time of Atto488 or TMR containing particles through the focus was determined using the temporal auto-correlation analysis of the fluorescence intensity fluctuations (Fig 2A). Here, the laser pulses of the two colors were delayed by ca. 20 ns with respect to each other, a technique called pulsed interleaved excitation (PIE) [32]. Using this additional timing information, it is possible to separate the signal not only by the detection channel, but also by the excitation source. Thus, the influence of spectral crosstalk was removed completely, resulting in correlation functions that are not biased by the presence of the other dye.

The diffusion coefficient inversely correlates with the particle size. This means that the diffusion coefficient of the dye conjugate increases when nucleotides are removed from the RNA construct over time. To quantify this degradation, an autocorrelation function (ACF) for two diffusing components was used to fit the data. In this simplified assumption, the slow component represents the full construct while the fast component corresponds to a digested dye-RNA fragment. The decrease in the amplitude of the slow component was taken as a measure for oligonucleotide degradation.

**Fig 3:**
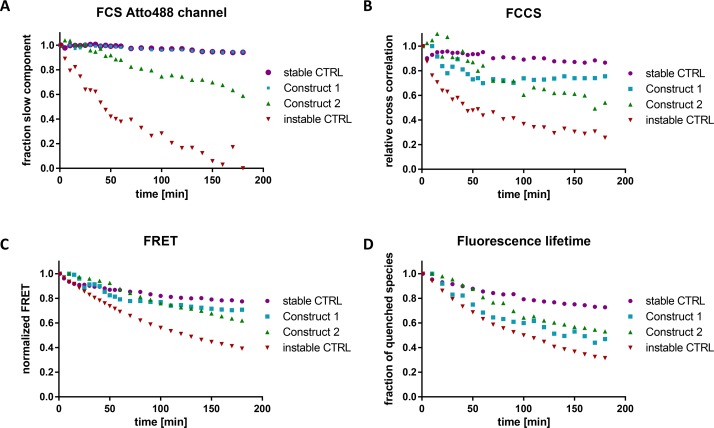
Evaluation of the degradation of the dual-labeled RNAs in cell extract by different techniques. The constructs were incubated in HeLa cell extract for 3 h and the degradation was monitored with a confocal microscope. The degradation was analyzed using FCS (A), FCCS (B) and FRET *via* intensity (C) and fluorescence lifetime (D). The curves were normalized to 1 for the initial data-point.

The degradation dynamics of the different RNAs can be nicely observed from the FCS signal of Atto488. While no change in ACF was observed for the stable control and construct 1, the instable control and construct 2 degraded with time (Fig 3A). Assuming a monoexponential decay, the half-life of construct 2 was 4.7 times longer than for the instable control (construct 2: 220 min, instable control: 47 min). This stabilizing effect is attributed to the chemically modified ends of construct 2 (Fig 1). A single cleavage event at the unmodified position of construct 1 might not be recognized by this technique, as the dyes still contain a relatively long stretch of modified RNA which contributes to the slow component in the fit.

Analysis of the ACF from FCS measurements of TMR provided similar results with construct 1 and the fully modified RNA strand being more stable than construct 2 and the unprotected RNA strand (S1 Fig). However, the change in the diffusion coefficient for the unprotected RNA strand was not as large as expected. This could be due to the association of the TMR with cellular proteins, as FCS measures only the mobility of the probe. The FCS measurements of TMR showed a high variation in diffusion coefficients of the slow component between the different constructs, suggesting that aggregation may play a crucial role. To avoid the complications of a purely FCS based analysis, we also performed a fluorescence cross-correlation analysis on the same data.

#### Fluorescence cross-correlation spectroscopy

In FCCS, the fluorescence intensity fluctuations in one channel (corresponding to Atto488) are correlated to the fluctuations in the other channel (corresponding to TMR). Hence, when a RNA carries both labels, a cross-correlation signal will be observed. A single cleavage event leads to a complete loss in the cross-correlation amplitude, which makes this technique very sensitive (Fig 2B). As with the dual-color FCS experiments, the use of PIE during FCCS experiments [32, 40] allows for the complete removal of spectral cross-talk and thus further increases the sensitivity of the technique. This method is less biased towards cellular protein association compared to FCS, as the cross-correlation does not depend on the size of the construct. The FCCS results were consistent with FCS, with the exception that construct 1 revealed a slight degradation compared to the stable control, which is in accordance with the increased sensitivity of FCCS (half-life of 610 min). As determined by this technique, the half-life of construct 2 (172 min) was 1.9 times longer than the half-life of the instable control with 89 min (Fig 3B and S1 Table).

#### Förster resonance energy transfer

Another measure of dual-labeled RNA integrity is FRET, which can be evaluated based on fluorescence intensity ratios or *via* the fluorescence lifetime of the donor dye. Non-radiative energy transfer from the donor to the acceptor dye depends on their spatial separation and can be observed for distances up to 10 nm. Hence, the intact double-labeled RNA should give a significantly higher emission in the red channel (TMR) and a reduced lifetime for the donor upon excitation of Atto488 than the cleaved construct (Fig 2C and 2D). Similar to FCCS, this technique is sensitive to a single cleavage event. Intensity based FRET is calculated from the ratio of the red signal after Atto488 excitation to the sum of red and green fluorescence after Atto488 excitation, corrected for spectral crosstalk of Atto488 into the acceptor channel and direct excitation of TMR. A parameter that was not accounted for was incomplete labeling of the RNA and possible degradation of the RNA before the experiment. However, since the RNA degradation in cell extracts was expected to be exponential, these parameters would only change the initial values and not affect the rate constants. FRET also provides information on the conformation of the RNAs. As transitions between coiled and stretched conformations change the distance between the dyes, the measured FRET efficiencies depend on the conformational state. Furthermore, FRET measurements can be sensitive to artifacts from pH or aggregation dependent quenching of the dyes.

The results from the FRET experiments show that the instable control is degraded the fastest with half-lives of 102 min and 128 min for measurements based on lifetime and intensity, respectively. As expected, construct 1 was more stable than construct 2 for the intensity measurements (half-life construct 1: 331 min, half-life construct 2: 239 min). For the lifetime based analysis, the stability-difference between the two constructs was insignificant (half-life construct 1: 149 min, half-life construct 2: 170 min). Interestingly, the stable control revealed a slight decrease in FRET efficiency over time (half-life: 401 min for lifetime based and 564 min for intensity based FRET) (Fig 3C and 3D, S1 Table). As no degradation is visible when evaluated by FCS or FCCS, we speculate that this is due to interactions with cellular components affecting the conformation of the construct or the lifetime of the Atto488.

Taken together, the results obtained from the different read-outs show that the extent of modification of the construct strongly correlates with its stability in cell extracts. Highly sensitive data for cleavage of just a single nucleotide can be obtained by FCCS. FRET provides additional information on the conformational state of the RNA while FCS detects association with cellular components. Applying only a single technique might lead to misinterpretation of the data, as artifacts like quenching, conformational changes and aggregation might be mistaken for stability related issues.

### Measurements in cells

#### Transfection

**Fig 4:**
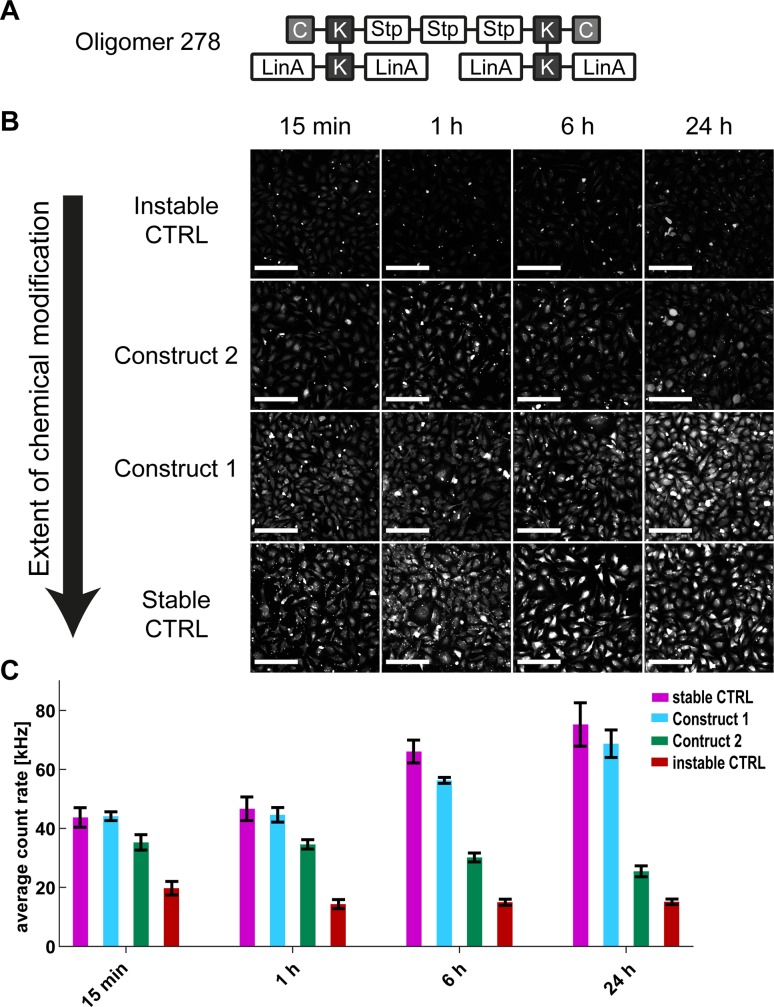
Fluorescence intensities of HeLa cells in culture after transfection with oligomer *278*. (A) A U-shaped, sequence defined cationizable lipo-oligomer ***278*** for complexation of the dual-labeled RNAs (C: cysteine, K: lysine, Stp: succinoyl-tetraethylene pentamine, linA: linoleic acid). (B) Fluorescence intensity images of the HeLa cells, 15 min, 1 h, 6 h and 24 h after transfection of the four different modifications patterns. The contrast level is equal for all images. The scale bar represents 200 μm. (C) Average fluorescence count rate of the cells at the different conditions shown in (B). The error bars represent the standard deviation of three independent measurements.

After having elucidated the fate of the chemically modified oligonucleotides in cell extracts, the next question was if the results hold true when the RNAs are transfected directly into HeLa cells. The sequence defined cationic oligomer ***278*** [29, 30] was selected as a carrier, as it displays fast cellular uptake, which is indispensable for a time course degradation experiment (Fig 4A). HeLa cells were incubated for 15 min with polyplexes formed using oligomer ***278*** and the respective modified oligonucleotide. Non-bound polyplexes were washed away from the cells followed by an additional incubation at 37°C. Biological processes were stopped at different time points by fixation with 4% paraformaldehyde and the cells were examined under a confocal microscope.

#### Fluorescence intensity

The fluorescence intensities of the four modification patterns were investigated after 15 min, 1 h, 6 h and 24 h (Fig 4B and 4C). We observed that the polyplexes are not immediately released after endosomal uptake. In fact, the RNA constructs continuously enter the cytosol over a longer time span. This accumulation is counteracted by RNA degradation and subsequent depletion from the cells. The fluorescence intensities that we observe for the four constructs at different time points reflect this balance (Fig 4C). In our measurements, the increase of the fluorescence intensity correlates with the extent of modification and, for the two most stable constructs, also with the duration of the incubation time after the transfection. For the less stable patterns, even a slight decrease in RNA concentration was observed over time. Especially the instable control shows very low fluorescence intensity at all time points. After the construct is taken up, it is almost immediately degraded by RNases. Since the dyes are no longer coupled to the RNA, they are not retained in the cells, resulting in a low steady-state concentration.

**Fig 5:**
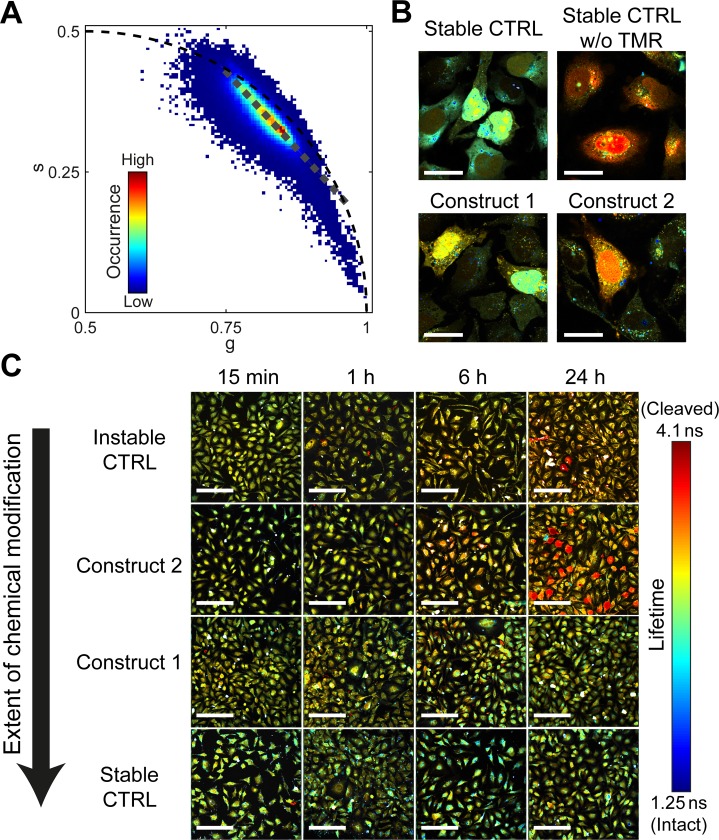
Phasor FLIM analysis in cultured HeLa cells. (A) The phasor histogram of images shown in panel B. The grey dotted line indicates the axis used for color-coding the FLIM images in (B) and (C). (B) FLIM images 24 h after transfection of the stable control RNA, construct 1, construct 2 and the stable control RNA without TMR in cultured HeLa cells. The scale bar is 30 μm. (C) FLIM images for all measured constructs and time points. These measurements are the same as those shown in Fig 4. The scale bar is 200 μm.

Taking a closer look at the cells, one can distinguish between two populations: cells showing a high fluorescence intensity in the nucleus and cells showing a higher fluorescence intensity in the cytosol (Fig 5B, S2 and S3 Figs). The cells that show a strong nuclear translocation of the constructs also have a brighter total intensity. This nuclear translocation is not present for the instable control as degradation dominates over other cellular processes.

#### Fluorescence lifetime in cells

Using FLIM, we can gather information regarding the integrity of the RNA constructs at different locations within the cells. Images were collected from cells that had been incubated at 37°C for 15 min, 1 h, 6 h and 24 h after transfection using oligomer 278 as the carrier. Due to quenching *via* FRET, Atto488 in intact constructs shows a reduced fluorescence lifetime compared to that in cleaved RNA fragments. Thus, the lifetime can be used as a read-out for the progress of RNA degradation. The image is scanned pixel by pixel and the lifetime at each spot is determined using the phasor approach to FLIM (Fig 5A and 5B).

The phasor approach is a fit-free way of analyzing lifetime data in the Fourier space by utilizing certain rules that simplify the analysis[41, 42]. The first of these rules is the fact that all purely mono-exponential decays lie on the universal circle centered at (0.5,0) with a radius of 0.5. The exact position on the circle is determined by the fluorescence lifetime, with short decays lying close to the (1,0) point while long lifetimes are closest to the origin. The second important rule is that mixtures of different lifetime species result in a phasor that is a linear combination of the two species. The vector is intensity-weighted meaning that any mixture of two lifetimes will lie on a straight line connecting the phasors of the pure species. Knowing the end positions of this line makes it possible to calculate the fractional contributions for any unknown mixture. In the phasor plot, the combined FLIM data of all measurements and patterns show a distribution along a line connecting the mono-exponential decays at 4.1 ns (corresponding to unquenched Atto488) and at 1.25 ns (corresponding to the intact construct showing FRET) (Fig 5A).

**Fig 6:**
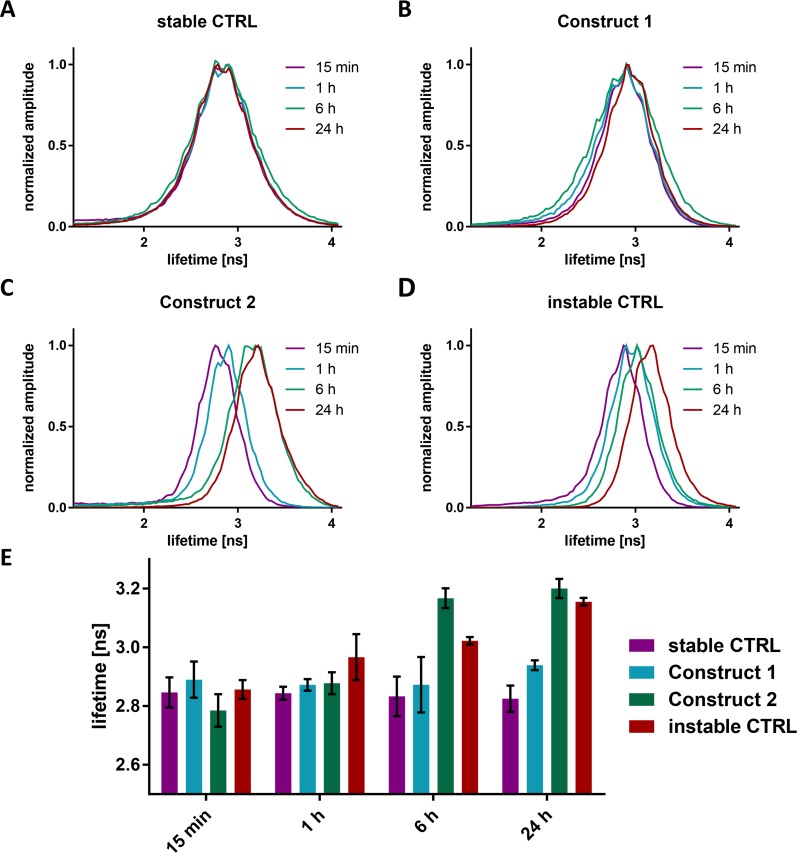
Quantification of the fluorescence lifetime measurements. (A-D) Distribution of the pixels along the line connecting the mono-exponential decays at 4.1 ns and at 1.25 ns in the phasor plot for the four modification patterns. (E) Summary of the average fluorescence lifetimes of the cell populations shown in panels A-D using a Gaussian fit to the distribution. The error bars represent the standard deviations of three independent measurements.

However, there are other sources of fluorescence quenching that need to be considered. The first one is quenching in densely packed particles (vesicles or polyplex aggregates). These regions are recognizable as small bright spots in the images and form a tail towards very short lifetimes in the phasor plot (S3 Fig). In constructs without an acceptor dye, quenching in these spots is still noticeable, but much reduced. This suggests that increased FRET between the densely packed RNAs is the main quenching source, but that self-quenching by the dyes or quenching from the carrier oligomer may also play a role. This is also noticeable with intensity based FRET (S4 Fig). Using an upper intensity threshold, these aggregates can be easily filtered out and removed from further analysis. As FRET is highly sensitive to the distance between the donor-acceptor dye pair, changes in the conformation of the RNA will also result in differences in the FRET efficiency and consequently in changes of the fluorescence lifetime. In order to investigate the degradation of the RNAs, the distribution of the pixels of the different patterns at different time points were plotted (Fig 5C, Fig 6A–6D) and the average value was extracted using a Gaussian fit (Fig 6E). The pixel positions along the line (i.e. the average pixel lifetimes) can also be depicted in the images using a color code (see Fig 5B and 5C). Blue represents a short fluorescence lifetime corresponding to an intact construct. Red represents a long lifetime indicating a degraded oligonucleotide.

In order to distinguish short lifetimes that originate from FRET and represent a high construct integrity from unspecific quenching effects, we included an additional control sample. Here, the stable control pattern was conjugated only to Atto488. Since no FRET can occur, any differences in lifetime observed are due to unspecific quenching. As expected, the fluorescence lifetime was long in the cytosol as well as the nucleus after 1 h and 24 h (S5 Fig). Nevertheless, plenty of small bluish dots could be observed in the cytosol. These spots are visible for all constructs and most probably possess a high density of Atto488 leading to quenching through aggregation (S3 and S4 Figs). Possible sources are endosomes that have not yet released the constructs or polyplexes that have not disassembled. Consequently, as soon as the oligonucleotides are liberated into the cytosol or the nucleus, a decreased lifetime can be assigned to FRET originating from an intact construct.

The dual-labeled stable control and construct 1 do not show any change in lifetime over the whole time course of 24 h (Fig 5C rows 3 and 4). This lack of degradation also explains the strong accumulation of the constructs within the cells. However, taking into account the fact that construct accumulation is a little stronger for the stable control, we assume there is a slight degradation of construct 1, which increases the ability of the cells to dispose of the oligonucleotide. Considering the fact that the cells are constantly fed with intact constructs from endosomes and aggregates, it is not surprising that the lifetime experiment reveals no significant differences (Fig 6A and 6B) as, at any point, the amount of degraded RNAs is negligible compared to that of the intact constructs. Construct 2 nicely shows an increase in lifetime over the time course, corresponding to degradation of the construct. Especially highly fluorescent cells have a long lifetime, originating from accumulation of the degraded oligonucleotide (Fig 6C). For the instable control, the observed fluorescence lifetime increases slower than for the more stable construct 2 (Fig 6C and 6D). However, considering the very low fluorescence intensity of the control, it is most likely that the oligonucleotides are degraded and depleted from the cells so fast that the lifetime values mostly originate from intact constructs that have just been released into the cytosol. In contrast, construct 2 Atto488 still has a stabilized four nucleotides stretch of RNA attached, which is not as easily expelled from the cells as free dye (Figs 5C and 6E). This is supported by the fact that the fluorescence intensity of construct 2 images is significantly higher than that of the instable control images.

In general, the measured lifetime values can be explained by a balance originating from the release of intact RNA, its degradation and subsequent depletion from the cells. Longer stretches of nucleotides accumulate in the cells and are transported into the nucleus when the cells are efficiently transfected. With decreasing length of the stabilized RNA stretch, depletion from the cells is favored.

## Conclusion

The stability of short single-stranded RNA oligonucleotides modified with phosphorothioates, 2’-O-Me and 2’-F was compared in cellular extracts and in cultured cells. By evaluating the degradation with FCS, FCCS and FRET (both intensity and fluorescence lifetime based) in cell extracts, we could conclude that each methodology provides distinct information on the behavior of the two-dye labeled constructs, which is indispensable to understand the fate of those RNAs. With FCCS, we monitor the integrity of the connection between the two dyes with very high sensitivity. Additional information on interactions with cellular components are detected by FCS, but at the expense of sensitivity for RNA degradation. FRET is not only sensitive towards construct cleavage, but can also detect conformational changes as FRET efficiency depends on the distance between the two dyes. We needed to utilize all techniques to minimize biases in the analysis from unspecific effects like aggregation, interactions with cellular components, quenching or conformational changes. Taking the results from the four techniques together, the non-modified oligonucleotide was, on average, degraded 2.2 times faster than the construct with the modified ends and 8.3 times faster than the almost completely modified construct 1. A completely stabilized stable control was not degraded in cell extracts. Even the short modification at the ends of construct 2 already have a significant effect on RNA stability in the cell extract. This is understandable, as the stabilization of the 3’- and 5’-ends interferes with exonucleolytic degradation of the RNA and hence increases the survival time of the construct. Modifications in the center regions additionally interrupt cleavage by endonucleases, further increasing the stability of the other constructs.

These findings are in good agreement with previous works on chemically modified RNAs[43].

Fluorescence lifetime measurements in cells in culture revealed interesting information on modification dependent integrity and localization of the oligonucleotides. We can conclude that non-stabilized single-stranded RNA oligonucleotides are degraded before a significant accumulation of the constructs can occur. For the stabilized constructs, on the other hand, intracellular fluorescence intensity increased with the extent of modification. Construct 2, with only a few modifications, showed no continuous increase in fluorescence intensity, but the equilibrium concentration was significantly higher than for the instable control. For construct 1 and the stable control, however, the release of the constructs into the cytosol far exceeded their degradation and depletion, leading to an accumulation over time. The short, non-modified region in construct 1 slightly decelerated the accumulation within the cells. Furthermore, in cells displaying a high fluorescence intensity, the oligonucleotides were transported into the nucleus. A high transfection rate is a prerequisite for the domination of the nuclear translocation mechanism over the depletion mechanism.

The lifetime of the constructs in living cells can be explained by a balance between release of the intact constructs from endosomes and polyplexes, and degradation by RNases and subsequent depletion of the fluorophores from the cells. The difference of construct 1 and the stable control observed for the fluorescence intensities has no significant effect on the lifetime measurements, since the small contribution of the cleaved constructs is drowned out by the majority of intact RNAs. Construct 2 transfected cells reached a plateau in lifetime after 6 h with a mean fluorescence lifetime that is ca. 0.35 ns longer than for the stable control. The slower increase in the fluorescence lifetime over time for the instable control can be attributed to the fast complete degradation and depletion of the dyes from the cells directly after their release into the cytosol. The lifetime values mostly originated from the few intact constructs immediately after their liberation.

Taken together, considering also the results from the cell extract measurements, we conclude that a fully PS modified backbone and alternating 2’-O-Methyl and 2’-Fluoro modifications provide complete resistance towards nuclease activity for single-stranded oligonucleotides under the experimental conditions. A single unmodified region accelerates degradation and reduces accumulation significantly, but still shows highly increased RNase resistance when compared to non-modified RNA. Stability decreases with the length of the stretch of unmodified nucleotides as demonstrated by the difference between construct 1 and construct 2.

With FLIM, we obtain information on the endosomal release, liberation from polyplexes and localization dependent stability of any double-labeled construct after its transfection. Hence, this technique can be a useful tool to understand more about the behavior of transfected oligonucleotides and their dependency on different chemical modification patterns. The information on localization-dependent integrity and availability can be used to figure out the bottlenecks of oligonucleotide delivery and help to specifically improve the functionality of a carrier.

## Supporting information

S1 Method Intensity FRET analysis. (PDF)

S2 Method Lifetime FRET analysis. (PDF)

S3 Method FCS and FCCS analysis. (PDF)

S4 Method FLIM analysis. (PDF)

S1 Fig Evaluation of the degradation of the dual-labeled RNAs in cell extracts by FCS on the TMR signal. (PDF)

S2 Fig Nuclear translocation of the dual-labeled RNAs in HeLa cells. (PDF)

S3 Fig Quenching of polyplexes. (PDF)

S4 Fig intensity based FRET images. (PDF)

S5 Fig Fluorescence lifetime imaging microscopy of the completely stabilized oligonucleotide conjugated with Atto488 only. (PDF)

S1 Table Half-lives of the differently modified constructs in HeLa cell extracts. (PDF)
