# Supplementary material for: Monitoring integrity and localization of modified single-stranded RNA oligonucleotides using ultrasensitive fluorescence methods
Source: PLoS One. 2017 Mar 9;12(3):e0173401. doi: 10.1371/journal.pone.0173401 (PMC5344492; doi:10.1371/journal.pone.0173401)
Supplement: S1 Method — (PDF) [file pone.0173401.s001.pdf]

**S1 Method. Intensity FRET analysis**

For the intensity based FRET analysis, the FRET efficiency *E* was calculated according to equation S1:

$$E = \frac{(I_{GR} - \beta \cdot I_{GG} - \alpha \cdot I_{RR})}{\gamma \cdot I_{GG} + (I_{GR} - \beta \cdot I_{GG} - \alpha \cdot I_{RR})} \qquad \text{(S1)}$$

where $I_x$ denotes the fluorescence intensity in channel x. The individual channels were GG and GR for the green and red fluorescence after excitation with the 475 nm laser, respectively, and RR for the red signal after 565 nm excitation. $\alpha$ represents the direct excitation of tetramethylrhodamine (TMR) at 475 nm excitation and $\beta$ stands for the spectral cross-talk of Atto488 into the red channel. Using single dye controls, these values were determined to be 7.4 % for $\alpha$ and 13 % for $\beta$ for the setup and measurement parameters used for this manuscript. $\gamma$, the parameter accounting for the different detection efficiency in the green and the red channels could not be determined for the setup and was assumed to be equal to unity. However, varying the parameter within a reasonable range showed no significant effect on the measured degradation times. The FRET efficiency was calculated in 5 min intervals for the first hour and subsequently in 10 min intervals. The data was normalized to the initial data point.
