# Supplementary material for: Monitoring integrity and localization of modified single-stranded RNA oligonucleotides using ultrasensitive fluorescence methods
Source: PLoS One. 2017 Mar 9;12(3):e0173401. doi: 10.1371/journal.pone.0173401 (PMC5344492; doi:10.1371/journal.pone.0173401)
Supplement: S2 Method — (PDF) [file pone.0173401.s002.pdf]

S2 Method. Lifetime FRET analysis

For analyzing fluorescence lifetime based FRET, the phasor approach was used[37, 38]. Calculations were performed using the home written software package PAM. Here, each 10 min of the signal in the green channel was divided into 2500 individual segments and, for each segment, the phasor coordinates were calculated. To account for the instrument response function (IRF), the data were referenced using an Atto488 carboxylic acid solution. The division into 2500 was done in order to be able to remove the influence of bright aggregates on the measured fluorescence lifetime.

In order to be able to extract the fraction of molecules from the phasor positions, a simple two-component system was assumed. The first component consists of intact RNA exhibiting FRET and therefore a reduced fluorescence lifetime for the donor molecule, while the degraded RNA fragments constitute the second species showing no FRET signal and a longer fluorescence lifetime. During the experiment, ongoing degradation changes the ratio between the two components but the individual lifetimes stay the same. In the phasor plot, such a shift in fraction results in a straight line connecting the two base components. The relative contribution of the individual species can then be extracted from the exact position on this line.

In practice, the lifetimes of both the intact and degraded species were not known *a priori*. To get these values, a line was extrapolated from the trajectory of the phasor coordinates during the measurement. The two intersects of this line with the universal circle were assumed to be the phasors of the base components. The lifetimes associated with these components varied slightly between the different species, but where generally around 1.2 ns for the cleaved species and 3.5 ns for the intact species. The later corresponds well to the lifetime measured for the control with just the donor fluorophore. From the center position of the 2500 segments, the relative photon contribution of these components was calculated. Finally, this photon contribution was further corrected for the reduced brightness of the intact species due to FRET resulting in the fraction of intact RNA. The relative brightness was estimated from the ratio of the fluorescence lifetime.
