# Supplementary material for: Monitoring integrity and localization of modified single-stranded RNA oligonucleotides using ultrasensitive fluorescence methods
Source: PLoS One. 2017 Mar 9;12(3):e0173401. doi: 10.1371/journal.pone.0173401 (PMC5344492; doi:10.1371/journal.pone.0173401)
Supplement: S3 Method — (PDF) [file pone.0173401.s003.pdf]

S3 Method. FCS and FCCS analysis

The auto- and cross correlation curves were calculated from the photon traces using the software package PAM. In the first hour of the measurement, correlation curves were calculated for every 5 min, afterwards for every 10 min.

The auto-correlations were fit with a two-component free diffusion model, assuming a 3D Gaussian shape:

$$G_{ACF}(\tau) = \left(1 + \frac{T}{1-T} \cdot e^{-\frac{\tau}{t_T}}\right) \cdot \frac{\gamma}{(N_{Fast} + \varepsilon \cdot N_{slow})^2} \cdot \left(\frac{N_{Fast}}{\left(1+\frac{\tau}{t_{Fast}}\right)\cdot\sqrt{1+\frac{\tau}{p^2 \cdot t_{Fast}}}} + \frac{\varepsilon^2 \cdot N_{Slow}}{\left(1+\frac{\tau}{t_{Slow}}\right)\cdot\sqrt{1+\frac{\tau}{p^2 \cdot t_{Slow}}}}\right) + G(\infty) \quad \text{(S2)}$$

Here, T and t_T denote the dark state fraction and correlation time, respectively. N_i represents the average number of molecules in the focus for the slow or the fast component, while t_i denotes their diffusion times. γ is the shape factor of a 3D Gaussian profile and corresponds to 0.35355. The parameter p is the ratio between the axial and lateral focus size. To relate the amplitudes of the two components into number of molecules, the difference in molecular brightness of the two species has to be taken into account. Thus, the relative brightness ε, caused by quenching of the intact construct due to FRET needs to be used. It is estimated as the ratio of the lifetimes of the quenched and the unquenched states as given by the lifetime FRET analysis mentioned above.

The slow component was assumed to represent the intact construct, while the fast component corresponds to the degraded fragments. Based on the N_i, the fraction of intact RNA was calculated for the green channel (GG) and the red channel after 565 nm excitation (RR).

The cross-correlation curves were fit with a simplified model using only a single component and no dark state term:

$$G_{CCF}(\tau) = \frac{\gamma}{N_{CC}} \cdot \frac{1}{\left(1+\frac{\tau}{t}\right)\cdot\sqrt{1+\frac{\tau}{p^2 \cdot t}}} + G(\infty) \quad \text{(S3)}$$

Based on the N_i from the auto- and cross-correlation functions, the fraction of intact RNA was calculated according to:

$$F_{slow} = \frac{\sqrt{(N_{fast,GG}+N_{slow,GG})\cdot(N_{fast,RR}+N_{slow,RR})}}{\varepsilon \cdot N_{CC}} \quad \text{(S4)}$$

The parameter ε again corrects for the decreased brightness of the intact species.
