# Supplementary material for: Monitoring integrity and localization of modified single-stranded RNA oligonucleotides using ultrasensitive fluorescence methods
Source: PLoS One. 2017 Mar 9;12(3):e0173401. doi: 10.1371/journal.pone.0173401 (PMC5344492; doi:10.1371/journal.pone.0173401)
Supplement: S4 Method — (PDF) [file pone.0173401.s004.pdf]

S4 Method. FLIM analysis

The phasor analysis for experiments on fixed cells was done in much the same way as for the lifetime based FRET measurements in cellular extracts. Pixels with intensities below a threshold of 200 photons were omitted from further analysis, thus eliminating the influence of the regions around the cells and limiting the influence of auto-fluorescence. An upper threshold of 1000-2000 photons was also applied to remove RNA aggregates from the analysis. As described above, a line connecting the phasors of the degraded and the intact species was extrapolated. A histogram was generated from the positions of the individual pixel along that line (Fig 6 A-D) that was subsequently fit with a Gaussian curve to extract the average lifetime (Fig 6 E).
