# Supplementary material for: Monitoring integrity and localization of modified single-stranded RNA oligonucleotides using ultrasensitive fluorescence methods
Source: PLoS One. 2017 Mar 9;12(3):e0173401. doi: 10.1371/journal.pone.0173401 (PMC5344492; doi:10.1371/journal.pone.0173401)
Supplement: S1 Fig — (PDF) [file pone.0173401.s005.pdf]

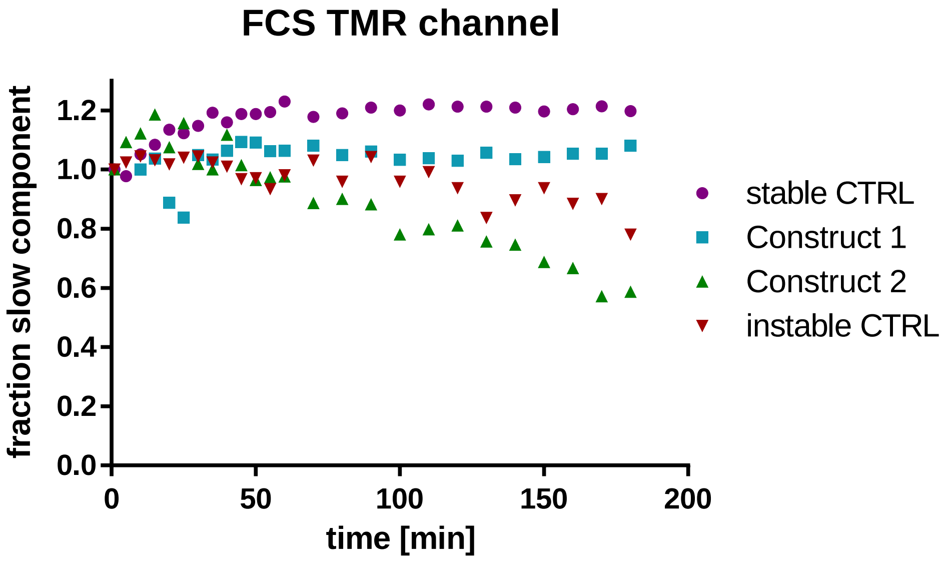

S1 Fig. Evaluation of the degradation of the dual-labeled RNAs in cell extracts by FCS in the TMR channel. The constructs were incubated in HeLa cell extracts for 3 h and the degradation was monitored via the decrease in the amplitude of the slow diffusing component using a confocal microscope.
