# Supplementary material for: Monitoring integrity and localization of modified single-stranded RNA oligonucleotides using ultrasensitive fluorescence methods
Source: PLoS One. 2017 Mar 9;12(3):e0173401. doi: 10.1371/journal.pone.0173401 (PMC5344492; doi:10.1371/journal.pone.0173401)
Supplement: S2 Fig — (PDF) [file pone.0173401.s006.pdf]

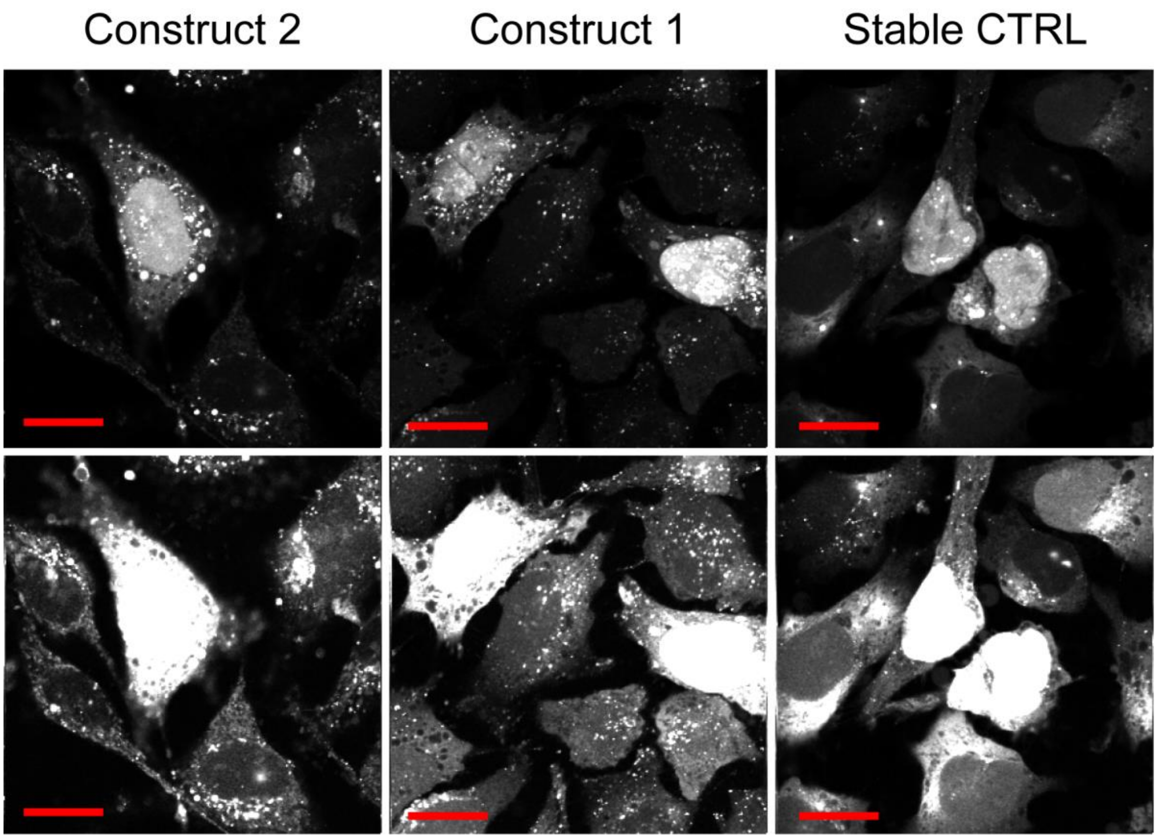

S2 Fig. Nuclear translocation of the dual-labeled RNAs in HeLa cells. Cells with increased RNA concentrations in the nucleus show also an increased overall brightness for the different stabilized constructs. The upper and lower images are the same. The contrast for the upper images is set to allow observation of the bright cells whereas, on the bottom images, the contrast is set to better visualize the dim cells. The scale bar is 20 µm.
