# Supplementary material for: Monitoring integrity and localization of modified single-stranded RNA oligonucleotides using ultrasensitive fluorescence methods
Source: PLoS One. 2017 Mar 9;12(3):e0173401. doi: 10.1371/journal.pone.0173401 (PMC5344492; doi:10.1371/journal.pone.0173401)
Supplement: S3 Fig — (PDF) [file pone.0173401.s007.pdf]

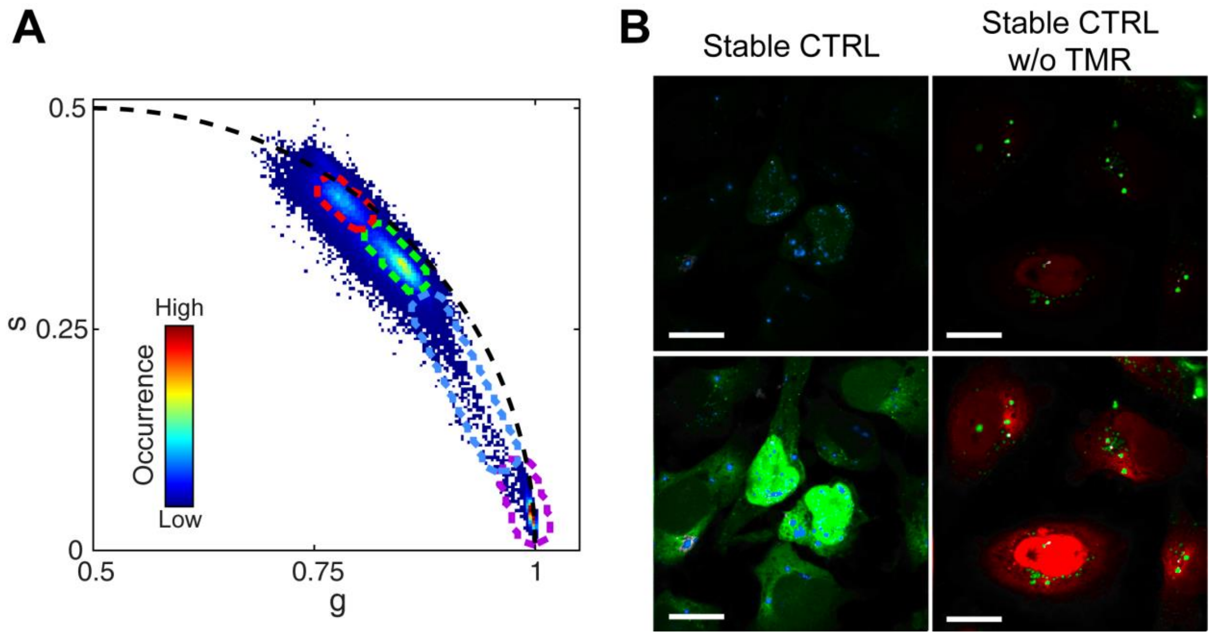

S3 Fig. Quenching in polyplexes. (A) Phasor histogram of the FLIM images shown in panel (B). The red, green and blue dotted ellipsoids were used to color code the individual pixels in panel (B). The magenta ellipsoid highlights the phasor of pure polyplexes (made with the stable control RNA) measured *in vitro*. (B) FLIM images of the stable control both with (left) and without (right) the acceptor dye (TMR) after 24 h incubation (upper panels). The contrast is set to visualize the shorter lifetime of the bright polyplex aggregates in the cells (lower panels). The image contrast was scaled to better visualize the cells. The scale bar is 20 µm.
