# Supplementary material for: Monitoring integrity and localization of modified single-stranded RNA oligonucleotides using ultrasensitive fluorescence methods
Source: PLoS One. 2017 Mar 9;12(3):e0173401. doi: 10.1371/journal.pone.0173401 (PMC5344492; doi:10.1371/journal.pone.0173401)
Supplement: S4 Fig — (PDF) [file pone.0173401.s008.pdf]

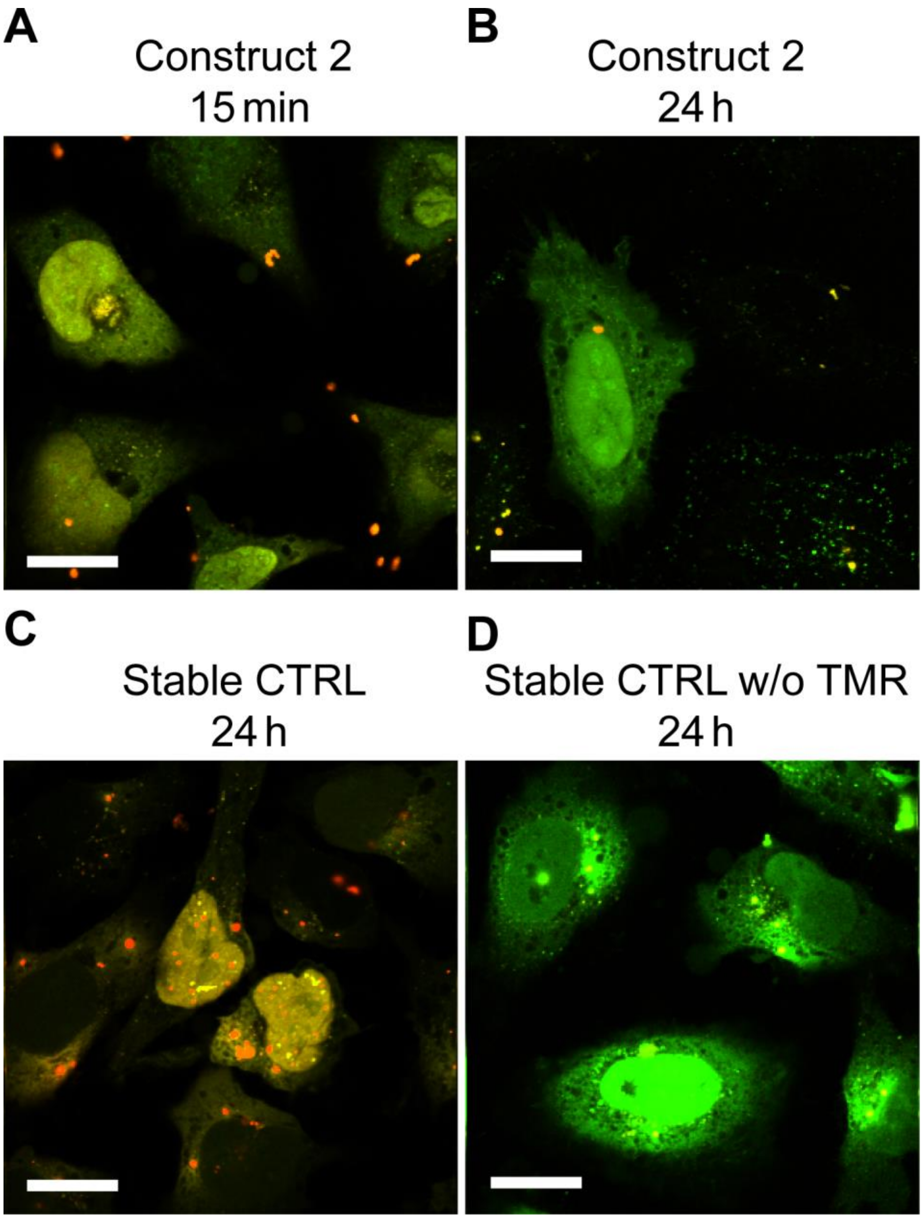

S4 Fig. Intensity based FRET images. Donor (green) and acceptor (red) signal after donor excitation is shown 15 min (A) and 24 h (B) after transfection for construct 2 and 24 h after transfection for the stable control with (C) and without (D) acceptor dye (TMR). The scale bar is 20 µm.
