# Supplementary material for: Monitoring integrity and localization of modified single-stranded RNA oligonucleotides using ultrasensitive fluorescence methods
Source: PLoS One. 2017 Mar 9;12(3):e0173401. doi: 10.1371/journal.pone.0173401 (PMC5344492; doi:10.1371/journal.pone.0173401)
Supplement: S5 Fig — (PDF) [file pone.0173401.s009.pdf]

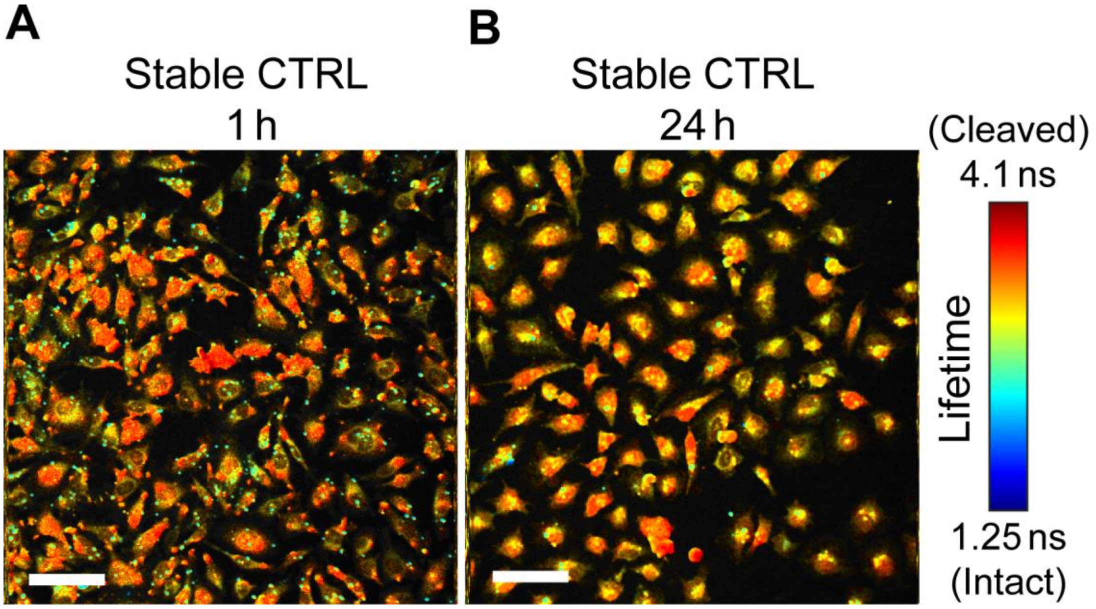

S5 Fig. Fluorescence lifetime imaging microscopy of the completely stabilized oligonucleotide conjugated only with Atto488. The fluorescence lifetime was measured 1 h and 24 h after transfection in HeLa cells. The scale bar is 100 µm.
