# Supplementary material for: Monitoring integrity and localization of modified single-stranded RNA oligonucleotides using ultrasensitive fluorescence methods
Source: PLoS One. 2017 Mar 9;12(3):e0173401. doi: 10.1371/journal.pone.0173401 (PMC5344492; doi:10.1371/journal.pone.0173401)
Supplement: S1 Table — (PDF) [file pone.0173401.s010.pdf]

S1 Table. Half-lives of the differently modified constructs in HeLa cell extracts

	FCS (blue channel)	FCCS	FRET (lifetime)	FRET (intensity)
**stable CTRL**	> 1000 min	> 1000 min	401 min	564 min
**Construct 1**	> 1000 min	610 min	148 min	331 min
**Construct 2**	220 min	172 min	170 min	239 min
**instable CTRL**	47 min	89 min	103 min	128 min
